# Effects of apolipoprotein B on lifespan and risks of major diseases including type 2 diabetes: a mendelian randomisation analysis using outcomes in first-degree relatives

**DOI:** 10.1016/S2666-7568(21)00086-6

**Published:** 2021-05-21

**Authors:** Tom G Richardson, Qin Wang, Eleanor Sanderson, Anubha Mahajan, Mark I McCarthy, Timothy M Frayling, Mika Ala-Korpela, Allan Sniderman, George Davey Smith, Michael V Holmes

**Affiliations:** Medical Research Council Integrative Epidemiology Unit; Medical Research Council Integrative Epidemiology Unit; University of Bristol, Bristol, UK; Clinical Trial Service Unit & Epidemiological Studies Unit, Nuffield Department of Population Health; University of Oxford, Oxford, UK; Systems Epidemiology, Baker Heart and Diabetes Institute, Melbourne, VIC, Australia; Computational Medicine, Faculty of Medicine, University of Oulu and Biocenter Oulu, Oulu, Finland; Medical Research Council Integrative Epidemiology Unit; Wellcome Centre for Human Genetics; Genetics of Complex Traits, University of Exeter Medical School, University of Exeter, Exeter, UK; Population Health Sciences, Bristol Medical School; Computational Medicine, Faculty of Medicine, University of Oulu and Biocenter Oulu, Oulu, Finland; Center for Life Course Health Research, University of Oulu, Oulu, Finland; NMR Metabolomics Laboratory, School of Pharmacy, University of Eastern Finland, Kuopio, Finland; Department of Medicine, McGill University, Montreal, QC, Canada; Medical Research Council Integrative Epidemiology Unit; Medical Research Council Integrative Epidemiology Unit; University of Bristol, Bristol, UK; Clinical Trial Service Unit & Epidemiological Studies Unit, Nuffield Department of Population Health; Medical Research Council Population Health Research Unit

## Abstract

**Background:**

Apolipoprotein B (apoB) is emerging as the crucial lipoprotein trait for the role of lipoprotein lipids in the aetiology of coronary heart disease. In this study, we evaluated the effects of genetically predicted apoB on outcomes in first-degree relatives.

**Methods:**

Data on lipoprotein lipids and disease outcomes in first-degree relatives were obtained from the UK Biobank study. We did a univariable mendelian randomisation analysis using a weighted genetic instrument for apoB. For outcomes with which apoB was associated at a false discovery rate (FDR) of less than 5%, multivariable mendelian randomisation analyses were done, including genetic instruments for LDL cholesterol and triglycerides. Associations between apoB and self-reported outcomes in first-degree relatives were characterised for 12 diseases (including heart disease, stroke, and hypertension) and parental vital status together with age at death. Estimates were inferred causal effects per 1 SD elevated lipoprotein trait (for apoB, 1 SD=0·24 g/L). Replication of estimates for lifespan and type 2 diabetes was done using conventional two-sample mendelian randomisation with summary estimates from genome-wide association study consortia.

**Findings:**

In univariable mendelian randomisation, genetically elevated apoB in participants was identified to lead to a shorter lifespan in parents (fathers: 0·89 years of life lost per 1 SD higher apoB in offspring, 95% CI 0·63–1·16, FDR-adjusted p=4·0 × 10^−10^; mothers: 0·48 years of life lost per 1 SD higher apoB in offspring, 0·25–0·71, FDR-adjusted p=1·7 × 10^–4^). The effects were strengthened to around 2 years of life lost in multivariable mendelian randomisation and were replicated in conventional two-sample mendelian randomisation (odds ratio [OR] of surviving to the 90th centile of lifespan: 0·38 per 1 SD higher apoB in offspring, 95% CI 0·22–0·65). Genetically elevated apoB caused higher risks of heart disease in all first-degree relatives and a higher risk of stroke in mothers. Findings in first-degree relatives were replicated in two-sample multivariable mendelian randomisation, which identified apoB to increase (OR 2·32 per 1 SD higher apoB, 95% CI 1·49–3·61) and LDL cholesterol to decrease (0·34 per 1 SD higher LDL cholesterol, 0·21–0·54) the risk of type 2 diabetes.

**Interpretation:**

Higher apoB shortens lifespan, increases risks of heart disease and stroke, and in multivariable analyses that account for LDL cholesterol, increases risk of diabetes.

**Funding:**

British Heart Foundation, UK Medical Research Council, and UK Research and Innovation.

## Introduction

Blood lipid concentrations have an important role in major vascular diseases, including coronary heart disease and stroke.^1^ LDL cholesterol is the conventionally used lipid trait in observational and genetic epidemiological studies^2,3^ and interventional trials.^4^ However, multiple sources of evidence show that the number of atherogenic lipoprotein particles, as estimated by apolipoprotein B (apoB), is a more accurate measure of the risk that lipoprotein lipids pose than the concentration of cholesterol that they contain, as estimated by either LDL cholesterol or non-HDL cholesterol.^5–8^ This has been shown in mendelian randomisation studies,^9–11^ the role of which has been amplified by the development of statistical tools, including multivariable mendelian randomisation.^12^ This approach permits the evaluation of the causal effects of specific traits while holding other traits constant, avoiding the introduction of collider bias.^13^

Although the crucial role of apoB in coronary heart disease is increasingly emerging,^5^ the comparative importance of apoB versus LDL cholesterol or triglycerides in other diseases remains less clear. In this study, we explore a broad repertoire of outcomes, taking advantage of events reported in first-degree relatives in UK Biobank. In studies using outcomes in first-degree relatives, the genetic instrument is constructed in individuals who share around 50% of their DNA with those in whom outcomes occur, which should halve the effect size. Associations identified in this way can provide insights into likely underlying causal relationships, which, if consistent with more conventional mendelian randomisation approaches, bolster confidence that the associations are real.

In this study, we explored the association between an apoB polygenic instrument and self-reported outcomes in first-degree relatives in participants of the UK Biobank for 12 diseases (including heart disease, stroke, hypertension, and Alzheimer’s disease) and parental vital status together with age at death. We assessed the rigour of these findings using multivariable mendelian randomisation by including genetic instruments for other major atherogenic lipoprotein lipids (LDL cholesterol and triglycerides), and comparing findings to previously published results.^10,12^ Finally, we sought replication of our findings for lifespan and type 2 diabetes using findings from large-scale genome-wide association study (GWAS) consortia, given that multivariable analyses using parental data showed evidence of an effect of apoB on these outcomes in the current study.

## Methods

### Datasets and study design

Data on lipoprotein lipids and disease outcomes in first-degree relatives were obtained from the UK Biobank study under application #15825. UK Biobank comprises approximately 500 000 participants who were recruited between 2006 and 2010 from 22 assessment centres across the UK. Details on sample handling and assays for lipoprotein lipids in UK Biobank have been described previously,^14^ as have general characteristics of the cohort, such as geographical regions and recruitment processes.^15^ Details on genotyping quality control, phasing, imputation, and association testing in UK Biobank are reported elsewhere.^16^ Ethical approval for this study was obtained from the UK Biobank Ethics Advisory Committee (approval number 11/NW/0382) and informed consent was collected from all participants enrolled in UK Biobank.

We sought to replicate estimates from UK Biobank using conventional two-sample mendelian randomisation. Data for lifespan were derived from a GWAS meta-analysis of cohorts with participants dichotomised according to whether they survived to an age corresponding to the 90th centile of lifespan.^17^ Data for type 2 diabetes were derived from the most recent large-scale European type 2 diabetes GWAS that included data from 32 studies and excluded participants from UK Biobank.^18^

### Procedures

Each lipoprotein lipid trait was normalised to have a mean of 0 and SD of 1 using inverse rank-normalisation. We did a GWAS on all UK Biobank participants with genotype and trait data after excluding individuals of non-European descent (based on K-means clustering of K=4) and standard exclusions (ie, withdrawn consent, mismatch between genetic and reported sex, and putative sex chromosome aneuploidy). BOLT-LMM version 2.3.2 (linear mixed model) software was used for the GWAS because it is robust to potential confounding caused by relatedness or population structure.^19,20^ Analyses were adjusted for age, sex, fasting status, and a binary variable denoting the genotyping chip used in individuals (the UK Biobank Axiom array or the UK BiLEVE array).

Genetic instruments for each lipid-related trait were derived by linkage disequilibrium clumping of GWAS results (appendix 1 tables S1–3). Independent genetic variants robustly associated with traits (p<5 × 10^–8^) were identified using a reference panel of 503 Europeans from phase 3 (version 5) of the 1000 Genomes Project.^21^ Clumping parameters of r^2^<0·001 and a distance of 1 megabase were used for instrument identification. Univariable sets of instruments for each lipoprotein trait were derived by applying linkage disequilibrium clumping on each trait’s GWAS results in turn. For multivariable instruments, we combined all GWAS results before applying linkage disequilibrium clumping to ensure independent instruments were identified among these correlated traits.

### Outcomes

UK Biobank participants were asked at enrolment whether their father, mother, or siblings had any of the following diseases: heart disease, stroke, high blood pressure, chronic bronchitis or emphysema, Alzheimer’s disease or dementia, diabetes, Parkinson’s disease, severe depression, lung cancer, bowel cancer, prostate cancer, or breast cancer. Additionally, individuals were asked whether their mother and father were still alive, and if not, at what age they had died. These questions were only asked of participants who indicated that they were not adopted as a child and regarded blood relations only. If there was any uncertainty over responses, participants were told to respond with “do not know”.

Each disease outcome for father, mother, and siblings was analysed by GWAS separately based on BOLT-LMM and the quality control pipeline described previously.^22^ Total numbers of cases and controls for each of these GWAS can be found in appendix 1 (table S4). Genetic estimates for all instruments were extracted from these GWAS results for two-sample mendelian randomisation analyses. In effect, we did analyses using a two-sample design (although the sample for the exposure and outcomes originated from the same dataset). Given that genetic variants for the instrument were identified from measurements in individuals separate to those that experienced disease (ie, the single nucleotide polymorphism [SNP] estimates for exposure and outcomes were in effect derived from different people, even though lipid traits were measured and data on outcomes were obtained from the same individual; appendix 2 p 2), the potential for overfitting should theoretically be reduced. However, to address issues of potential overfitting leading to false positives, we sought to replicate key findings, as described below, using data on outcomes from non-overlapping datasets.

### Statistical analysis

We calculated mean *F*-statistics to evaluate the instrument strengths in both the univariable and multivariable mendelian randomisation using the approximation described by Bowden and colleagues^23^ for the univariable analysis and the conditional *F*-statistic by Sanderson and colleagues^24^ for the multivariable analysis.

We investigated the effect of genetically predicted higher concentrations of apoB on each of the 38 first-degree relative outcomes (described in appendix 1 table S4) in turn using univariable mendelian randomisation. We define genetically predicted apoB as the levels of circulating apoB arising from genetic variation. Genetic effects on apoB and outcomes for each instrument were harmonised and initial analyses were done using the inverse variance weight (IVW) method. Proxy SNPs were not necessary for this analysis given that both exposure and outcome datasets were from UK Biobank. All univariable mendelian randomisation analyses were repeated for each outcome using LDL cholesterol and subsequently triglycerides as the exposure. Next, we did multivariable mendelian randomisation analyses to investigate the effects of apoB, LDL cholesterol, and triglycerides simultaneously on outcomes in first-degree relatives. This permitted us to estimate the direct effect of apoB (ie, while accounting for the genetically predicted effects of LDL cholesterol and triglycerides) and allowed us to investigate the comparative causal roles of atherogenic lipoprotein traits on lifespan and disease risk, as proxied by outcome data from first-degree relatives. In this Article, we use the term direct to describe the effect of a lipid trait on disease while accounting for either mediation or confounding by another trait included in the model.

Estimates from mendelian randomisation are presented as odds ratios (ORs) per 1 SD higher lipid trait. Effect estimates for the association of lipid traits with vital status and age at death of parents are also presented in this way because, by being obtained from UK Biobank individuals at study entry, data on parental vital status are effectively cross-sectional.

For effects in the univariable mendelian randomisation analysis that survived correction for false discovery rate (FDR) of less than 5% based on estimates from the IVW method, we applied the weighted median,^25^ weighted mode,^26^ and MR-Egger^27^ regression approaches. For multivariable analyses,^13^ we additionally did multivariable MR-Egger.^28^

To replicate the findings identified for lifespan and type 2 diabetes, we did a conventional two-sample mendelian randomisation analysis in which SNPs for exposures (apoB, LDL cholesterol, and triglycerides) were derived from UK Biobank participants and SNPs for lifespan (grouping study participants based on whether they survived to the age corresponding to the 90th centile) and type 2 diabetes were derived from DIAMANTE, a previous large-scale GWAS.^17,18^ The GWAS for lifespan used UK Biobank as a form of validation and the estimates we use here do not include UK Biobank. However, the latest release of data from DIAMANTE included type 2 diabetes cases from UK Biobank; since including these data might have led to overfitting of the mendelian randomisation estimate, we analysed type 2 diabetes GWAS estimates on individuals of European descent with UK Biobank data removed. We did conventional univariable IVW two-sample mendelian randomisation. We then fitted a multivariable mendelian randomisation model simultaneously including genetic instruments for apoB, LDL cholesterol, and triglycerides to explore their direct effects on lifespan and type 2 diabetes. We did a MVMR-Egger^28^ sensitivity analysis to evaluate whether the mendelian randomisation estimates were influenced by dose−response confounding of the genetic instruments.

Because the apoB effects on risk of type 2 diabetes might be confounded by adiposity and fat distribution, we additionally included body-mass index (BMI) and waist–hip ratio adjusted for BMI (WHRadjBMI) in the multivariable mendelian randomisation analyses for type 2 diabetes. 641 SNPs used in the instrument for BMI were derived from 461 377 UK Biobank participants using the same GWAS pipeline as described previously.^12^ 398 SNPs were used in the instrument for WHRadjBMI using a previous GWAS within UK Biobank.^29^ We also used the IVW, MR-Egger, and weighted median approaches in a univariable setting to investigate the potential bidirectional relationship between apoB and type 2 diabetes.

Because some outcomes (ie, prostate cancer and breast cancer) occur only or predominantly in men or women, we re-ran the GWAS stratifying by sex and subsequently derived genetic instruments and did mendelian randomisation analyses using the aforementioned methods. To quantify the extent to which coronary heart disease and type 2 diabetes mediated the effects of apoB on lifespan (selected because these were among the most reliable findings identified, and these diseases are recognised to be among the top ten causes of death globally, and therefore might be expected to mediate the relationship between apoB and lifespan), we constructed multivariable mendelian randomisation models that included apoB, type 2 diabetes, coronary heart disease, and lifespan.

Because first-degree relatives share around 50% of DNA, and because outcomes were reported multiple times among first-degree relatives, the exposure-to-outcome analyses are not truly independent and use of conventional Bonferroni approaches to address multiple testing would be overly stringent. We therefore used Benjamini-Hochberg FDR of less than 5% to guide our interpretation of results from the initial univariable mendelian randomisation analysis of apoB for all 38 outcomes. This strategy was used to highlight which outcomes to evaluate in further detail in all subsequent analyses. FDR corrections were also applied to all additional univariable and multivariable analyses using UK Biobank participant data for completeness.

BOLT-LMM software^19^ was used to do GWAS analyses and identify genetic instruments. The TwoSampleMR R package was used for all mendelian randomisation analyses.^30^ The ggplot2 R package was used to generate forest plots.^31^ Conditional *F*-statistics were generated using the MVMR R package.

### Role of the funding source

The funders of the study had no role in study design, data collection, data analysis, data interpretation, or writing of the report.

## Results

A median of 400 304 UK Biobank participants (range 273 111–454 999) reported information on prevalent diseases in first-degree relatives. A median of 361816 participants (361 199–364 661) reported data for their siblings, 423 692 (422 464–426 391) reported data for their mothers, and 400 687 (399 089–407 557) reported data for their fathers (appendix 1 table S4). 180 472 (39·7%) participants reported that their mothers were alive and 103 919 (23·1%) reported that their fathers were alive. The mean age at death among deceased parents was 75·7 years (SD 13·3) in mothers and 70·9 years (13·1) in fathers.

Across the diseases reported as occurring in first-degree relatives (table), the prevalence was similar in fathers and mothers, with siblings having the lowest prevalence of disease. Mothers were disproportionately affected by some diseases (eg, Alzheimer’s disease or dementia [36 048 cases; 8·6%] and severe depression [28 351; 6·7%]) compared with fathers (Alzheimer’s disease or dementia: 19 255 cases, 4·8%; severe depression: 15 430 cases, 3·9%). The prevalence of heart disease was highest in fathers (133 320 cases; 32·7%), with mothers (85 620 cases; 20·1%) and siblings (37 858 cases; 10·4%) being less affected. The prevalence of stroke was similar in fathers (62 810 cases; 15·6%) and mothers (60 880 cases; 14·3%) but lower in siblings (12 031 cases; 3·3%), whereas the prevalence of type 2 diabetes was similar in all first-degree relatives (38 850 cases, 9·7% in fathers; 40 091 cases, 9·5% in mothers; and 31073 cases, 8·6% in siblings).

Our apoB GWAS in the UK Biobank explained 10·4% of the heritability in this trait. Linkage disequilibrium clumping identified 229 apoB-associated SNPs with an *F*-statistic of 160. For multivariable mendelian randomisation, in which genetic instruments for apoB, LDL cholesterol, and triglycerides were analysed simultaneously, an additional 197 SNPs associated with LDL cholesterol (*F*-statistic of 167) and 411 SNPs associated with triglycerides (*F*-statistic of 121) were included in the analysis. In multivariable mendelian randomisation, the conditional *F*-statistics for apoB and LDL cholesterol were similar (37 and 34), and for triglycerides the *F*-statistic was 80. Thus, in both univariable and multivariable mendelian randomisation settings, our genetic instruments would not be considered weak.

**Figure 1:**
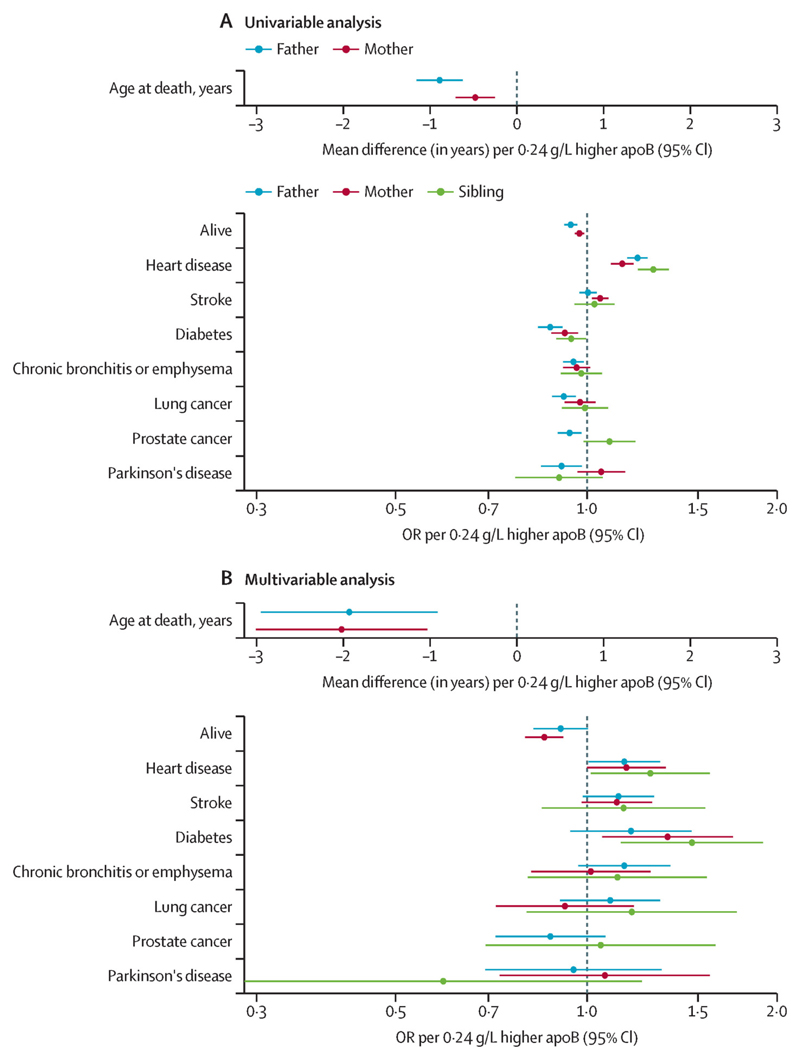
Univariable (A) and multivariable (B) mendelian randomisation estimates of genetically elevated apoB and risk of outcomes in first-degree relatives, including vital status and age at death Multivariable estimates represent the direct effects of apoB, adjusted for LDL cholesterol and triglycerides. ApoB=apolipoprotein B. OR=odds ratio.

Genetically elevated apoB was associated with a lower relative odds that an individual’s parents were alive (fathers: OR 0·94, 95% CI 0·92–0·97; mothers: 0·97, 0·96–0·99; figure 1A; appendix 1 table S5). Fathers were estimated to die 0·89 years (95% CI 0·63–1·16; FDR-adjusted p=4·0 × 10^–10^) earlier (corresponding to a mean of 10·7 months of life lost per 1 SD higher apoB in offspring) and mothers 0·48 years (0·25–0·71; FDR-adjusted p=1·7 × 10^–4^) earlier (corresponding to a mean of 5·8 months of life lost per 1 SD higher apoB in offspring). When taking into account the effects of LDL cholesterol and triglycerides in multivariable mendelian randomisation, these estimates for apoB increased to 1·94 years of life lost (0·91–2·96; FDR-adjusted p=0·0010) in fathers and 2·02 years of life lost (1·03–3·01; FDR-adjusted p=3·9 × 10^–4^) in mothers, with the inverse relative odds for being alive also becoming more pronounced (figure 1B; appendix 1 table S6).

In univariable mendelian randomisation, a higher risk of heart disease was evident in all first-degree relatives of individuals with genetically elevated apoB (fathers: OR 1·20, 95% CI 1·16–1·25, FDR-adjusted p=1·9 × 10^−21^; mothers: 1·14, 1·09–1·19, FDR-adjusted p=5·4 × 10^−9^; siblings: 1·27, 1·20–1·35, FDR-adjusted p=7·6 × 10^–16^). Mothers of individuals with genetically elevated apoB had a higher risk of stroke (1·05, 1·02–1·08, FDR-adjusted p=4·2 × 10^–4^; figure 1A). Estimates were similar, although less precise, in multivariable mendelian randomisation (figure 1B).

Parents of individuals with genetically elevated apoB had a lower risk of type 2 diabetes (fathers: OR 0·88, 95% CI 0·84–0·91, FDR-adjusted p=2·3 × 10^–8^; mothers: 0·92, 0·88–0·97, FDR-adjusted p=0·0042), whereas the decrease in risk was weaker in siblings (0·95, 0·90–0·998, FDR-adjusted p=0·082; figure 1A). In multivariable mendelian randomisation, the direction of effect was reversed: higher apoB was associated with elevated risk of type 2 diabetes (mothers: 1·34, 1·06–1·70, FDR-adjusted p=0·041), although the increase in risk was weaker in fathers (1·17; 0·94–1·46, FDR-adjusted p=0·24; figure 1B).

**Figure 2:**
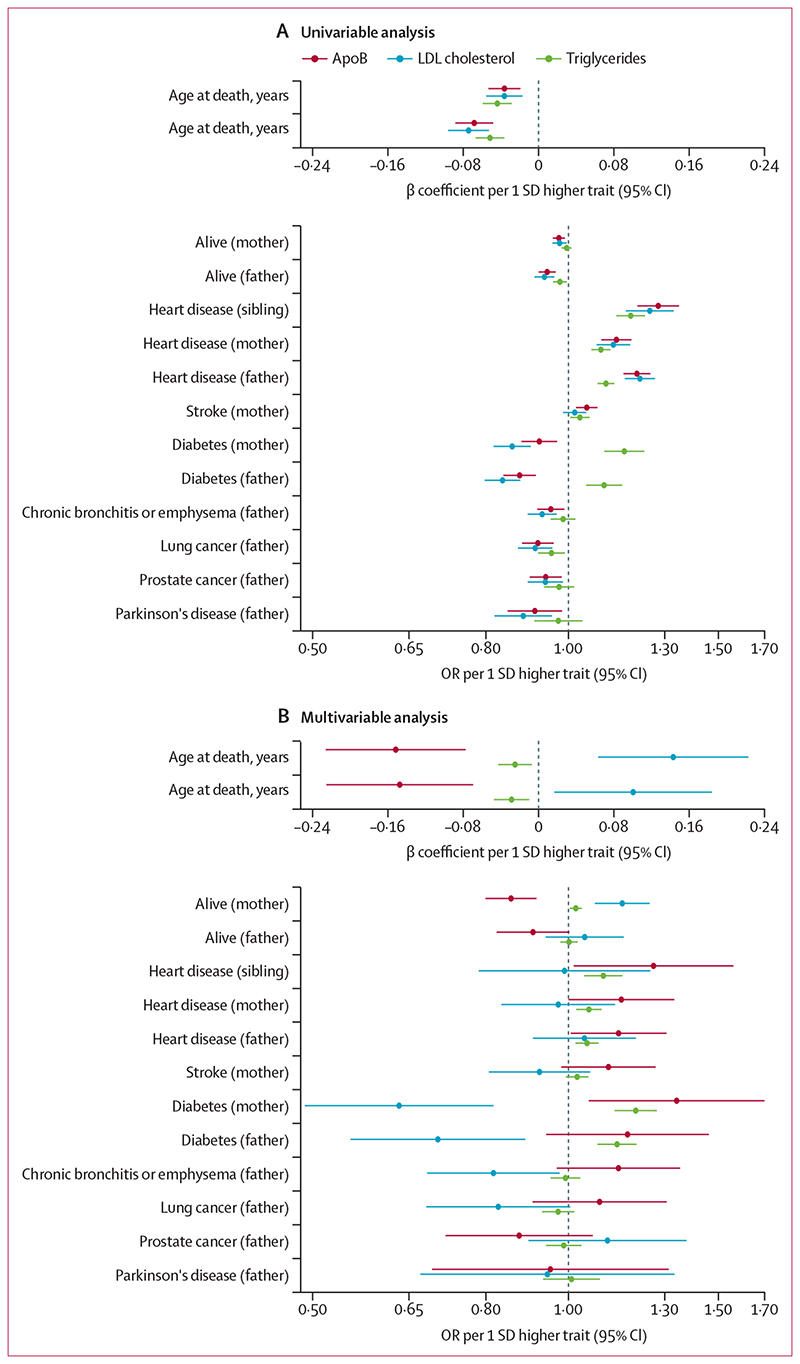
Univariable (A) and multivariable (B) mendelian randomisation estimates of association between genetically elevated apoB, LDL cholesterol, and triglycerides and risk of outcomes in first-degree relatives, including vital status and age at death Multivariable estimates represent the direct effects of each lipoprotein entity, adjusted for the other two traits. ApoB=apolipoprotein B. OR=odds ratio.

In univariable mendelian randomisation, the associations of LDL cholesterol with diseases in first-degree relatives were similar to those of apoB for all outcomes (figure 2A; appendix 1 table S7). However, effect estimates differed in multivariable mendelian randomisation. When taking into account apoB and triglycerides, higher LDL cholesterol was associated with an older age at death in both parents (fathers: increase of 1·31 years per 1 SD increase in LDL cholesterol, 95% CI 0·22–2·41, FDR-adjusted p=0·045; mothers: increase of 1·90 years, 0·84–2·96, FDR-adjusted p=0·0017) and a higher probability that mothers were alive (OR 1·16, 1·07–1·25, FDR-adjusted p=7·6 × 10^–4^). For fathers, the increase in the odds of being alive was weaker (1·04, 0·94–1·16, FDR-adjusted p=0·53; figure 2B; appendix 1 table S6).

By contrast, the univariable and multivariable estimates of the association of LDL cholesterol with risk of type 2 diabetes were directionally constant: higher LDL cholesterol was associated with a lower risk of type 2 diabetes, irrespective of whether genetic associations with apoB and triglycerides were included (fathers: OR 0·84, 95% CI 0·80–0·88, FDR-adjusted p=3·3 × 10^–13^; in univariable mendelian randomisation and 0·70, 0·55–0·89, FDR-adjusted p=3·3 × 10^–3^ in multivariable mendelian randomisation; mothers: 0·86, 0·82–0·90, FDR-adjusted p=2·7 × 10^–9^ in univariable mendelian randomisation and 0·63, 0·49–0·82, FDR-adjusted p=4·2 × 10^–4^ in multivariable mendelian randomisation; figure 2).

A positive relationship of LDL cholesterol with heart disease in parents and siblings (and with stroke in mothers) in univariable mendelian randomisation became weaker when accounting for apoB and triglycerides in the multivariable analysis, as did the inverse relationships with lung cancer, Parkinson’s disease, and prostate cancer (figure 2).

Estimates for triglycerides were generally robust to multivariable mendelian randomisation analyses (figure 2; appendix 1 table S8). Higher triglycerides were associated with an earlier age at death in both parents (fathers: 0·68 years of life lost, 95% CI 0·47–0·88, FDR-adjusted p=0·0072; mothers: 0·58 years of life lost, 0·38–0·79, FDR-adjusted p=0·015), but the magnitude of association was smaller than for apoB in multivariable mendelian randomisation. Similarly, positive associations between triglycerides and heart disease in parents and siblings in univariable mendelian randomisation were robust to multivariable mendelian randomisation, but the magnitudes of effect were attenuated. For type 2 diabetes, positive associations with triglycerides were evident in both univariable and multivariable mendelian randomisation analyses.

**Figure 3:**
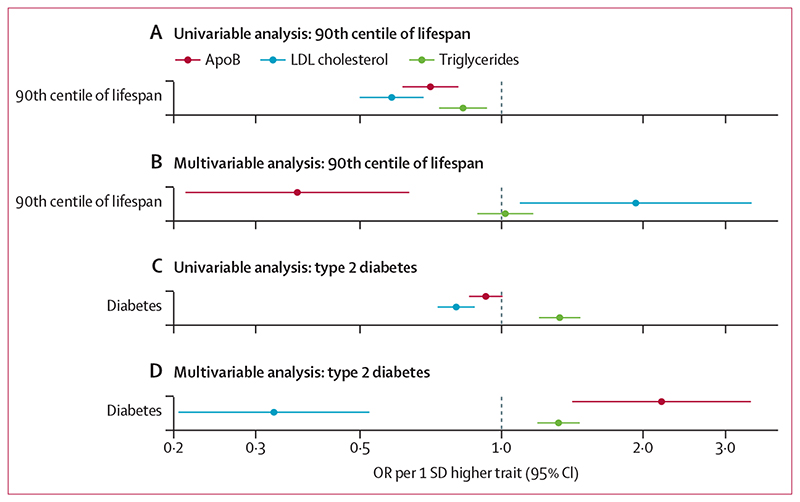
Univariable and multivariable estimates of genetically elevated apoB, LDL cholesterol and triglycerides with lifespan (A, B) and risk of type 2 diabetes (C, D) using two-sample mendelian randomisation Multivariable mendelian randomisation estimates represent the direct effects of each lipoprotein entity, adjusted for the other two traits. ApoB=apolipoprotein B. OR=odds ratio.

Given the findings for vital status and risk of type 2 diabetes in first-degree relatives, we sought to further elucidate these relationships using a conventional two-sample mendelian randomisation framework with data from two GWAS consortia: a GWAS of lifespan with 11 262 individuals surviving to the age corresponding to the 90th survival centile and DIAMANTE, including 55 927 cases of type 2 diabetes. The analyses of lifespan replicated our findings from age of death and vital status of parents (figure 3, appendix 1 table S9). ApoB was detrimental to survival (ie, higher apoB caused individuals to have a younger age of death than the age corresponding to the 90th survival centile) and this association was stronger when taking into account the effects of LDL cholesterol and triglycerides in multivariable mendelian randomisation. An increase in apoB by 1 SD in multivariable mendelian randomisation was associated with lower relative odds of surviving to the 90th centile of lifespan (OR 0·38, 95% CI 0·22–0·65). By contrast, the initial harmful effect of LDL cholesterol on lifespan in univariable mendelian randomisation was directionally reversed in multivariable mendelian randomisation. Likewise, triglycerides had a detrimental effect on lifespan in univariable mendelian randomisation, but the relationship became weaker in multivariable mendelian randomisation.

No clear effect of genetically predicted apoB on type 2 diabetes was identified in univariable mendelian randomisation. In contrast, higher LDL cholesterol caused a lower risk of type 2 diabetes (OR 0·84 per 1 SD higher LDL cholesterol, 95% CI 0·76–0·92) and higher triglycerides caused a higher type 2 diabetes risk (1·40 per 1 SD higher triglycerides, 1·26–1·55) in univariable mendelian randomisation. In multivariable mendelian randomisation, the estimate for triglycerides was largely unchanged (1·39 per 1 SD higher triglycerides, 1·25–1·54). However, the protective effect of LDL cholesterol against type 2 diabetes became more pronounced (0·34 per 1 SD higher LDL cholesterol, 0·21–0·54) and a strong positive association between apoB and type 2 diabetes risk emerged (2·32 per 1 SD higher apoB, 1·49–3·61; figure 3, appendix 1 table S10).

Repeating analyses using robust mendelian randomisation approaches for both univariable and multivariable mendelian randomisation led to generally consistent associations between apoB and risk of disease in first-degree relatives (appendix 1 tables S11–15, appendix 2 pp 3–6), with risk of type 2 diabetes in DIAMANTE (appendix 1 table S16, appendix 2 p 7), and with lifespan (appendix 1 table S9). The estimate of triglycerides and risk of type 2 diabetes was notable for being directionally opposite between IVW and MR-Egger in both univariable analyses for type 2 diabetes in parents (appendix 2 p 4) and in conventional mendelian randomisation in DIAMANTE (appendix 1 table S16, appendix 2 p 7). Of note, using multivariable MR-Egger and orientating SNPs so that they all associated with higher triglycerides led to an attenuation of the association between triglycerides and type 2 diabetes, suggesting that it might be explained by unbalanced horizontal pleiotropy (appendix 1 table S16). Excluding variants from in and around the *APOE* locus led to a marked attenuation of the relationship between genetic instruments for apoB and LDL cholesterol and risk of Alzheimer’s disease in mothers (appendix 1 tables S17, S18). Inclusion of BMI in the multivariable mendelian randomisation for type 2 diabetes in DIAMANTE had no notable effect on the direct causal effects of apoB, LDL cholesterol, or triglycerides (appendix 1 table S19, appendix 2 p 8), which was also the case when accounting for WHRadjBMI (appendix 1 table S20, appendix 2 p 8). We also applied univariable mendelian randomisation to evaluate evidence of a bidirectional association between apoB and type 2 diabetes (ie, whether genetic liability to type 2 diabetes affects apoB levels). We identified weak evidence of an effect of type 2 diabetes liability on apoB in this analysis using genetic instruments derived from DIAMANTE based on the IVW and MR-Egger methods, whereas the weighted median estimate was directionally opposite to the multivariable mendelian randomisation estimate between apoB and type 2 diabetes (appendix 1 table S21). We did sex-specific analyses for diseases only occurring in women and men and found that the results were largely consistent with the sex-agnostic genetic instrument (appendix 1 table S22). Multivariable mendelian randomisation estimated that coronary heart disease and type 2 diabetes explained between 25% and 40% of the relationship between apoB and lifespan (appendix 1 table S23).

## Discussion

Our findings implicate apoB in several major diseases, including heart disease, stroke, and diabetes. Importantly, a higher apoB was associated with a shorter lifespan in parents, as shown by both a lower odds that an individual’s parents were alive and a younger age at their death. Inclusion of LDL cholesterol and triglycerides in multivariable mendelian randomisation strengthened these effects, suggesting that apoB was the predominant driving influence on lifespan among these three lipid traits. All of these findings were replicated using an independent dataset, verifying their robustness. Taken together, our findings show that reductions in apoB should be the primary goal of lipid lowering, because not only does this lead to lower risk of common diseases such as heart disease and stroke, but also a reduction in apoB prolongs life by a period of months to years. Importantly, the effect estimates we report in this study are likely to be diluted due to the nature of the exposure and outcome. The real magnitudes of effect, in terms of duration of life lost due to elevated apoB, are likely to be greater, as indicated in the effect estimates from the GWAS of 90th centile of survival. Our findings further strengthen the suggestion that it is the number of circulating apoB particles, rather than their lipid content, that is the critical element for atherogenesis, manifested as coronary heart disease and ischaemic stroke.^5,6,8^ The cholesterol within LDL particles does play a causal role in atherogenesis but it is within the physiological framework of the trapping of apoB particles within the arterial wall.

The findings for type 2 diabetes are intriguing. In naive univariable mendelian randomisation analysis, a higher LDL cholesterol was associated with a lower risk of type 2 diabetes, a finding that was strengthened with multivariable mendelian randomisation analysis. By contrast, a higher apoB was associated with a lower risk of type 2 diabetes in univariable mendelian randomisation, but in multivariable mendelian randomisation, a strong, positive association between apoB and risk of type 2 diabetes emerged. This finding was replicated using data from DIAMANTE, providing further evidence of a potentially direct causal relation between apoB and type 2 diabetes.

The majority of apoB particles in plasma are LDL particles, which are cleared most efficiently by the LDL pathway. Although the liver is the major site of LDL clearance, peripheral tissues, such as the pancreas, also have LDL receptors and clear LDL particles (appendix 2 p 9).^32^ Moreover, experimentally increasing cholesterol uptake or reducing cholesterol efflux produces islet cell dysfunction with reduced insulin secretion and cell proliferation.^33–38^ All of these findings are consistent with the hypothesis that increased uptake of LDL particles by the LDL pathway could contribute to the pathogenesis of type 2 diabetes.

LDL particles tend to be depleted in cholesterol when LDL particles are overproduced.^6,39^ This is the scenario approximated by our multivariable mendelian randomisation analysis when apoB is increased and LDL cholesterol kept constant. In this situation, clearance of LDL particles by the LDL pathway is increased, with the result that delivery of LDL particles to pancreatic islet cells could be increased (appendix 2 p 10). By contrast, decreased LDL clearance via the LDL pathway is characterised by LDL particles that are cholesterol-enriched.^6,39^ This is the scenario approximated by our multivariable mendelian randomisation analysis when LDL cholesterol is increased and apoB kept constant, and the risk of type 2 diabetes is reduced. In support of our findings is the reduced incidence of diabetes in familial hypercholesterolaemia,^40^ a disorder characterised by markedly reduced uptake of LDL particles and the cholesterol they contain via the LDL pathway.

In-vitro and mendelian randomisation analyses have shown that increased activity of the LDL pathway related to proprotein convertase subtilisin/kexin type 9 (PCSK9) deficiency is associated with an increased incidence of diabetes.^36–38,40–43^ The finding that the incidence of dysglycaemia was higher in individuals with cholesterol-depleted apoB particles and lower in individuals with cholesterol-enriched apoB particles is also consistent with our hypothesis.

Alternatively, increased uptake of LDL particles by adipocytes has been posited by Faraj^44^ to induce adipose tissue dysfunction with an increased inflammatory response, resulting in reduced insulin sensitivity. Faraj^44^ has also suggested that cytotoxic injury to adipocytes due to increased uptake of LDL might be a mechanism by which statins and PCSK9 inhibitors, agents which increase activity of the LDL pathway, could be diabetogenic. Accordingly, we hypothesise that increased uptake of LDL particles might injure adipocytes or islet cells or both, which might explain the strong positive association between apoB and the risk of diabetes that was observed in this study.

The resilience of our findings to inclusion of adiposity measured by BMI and WHRadjBMI (indexing total and visceral adiposity, respectively) in the multivariable mendelian randomisation suggests that the associations we identify with risk of type 2 diabetes are not confounded by upstream adiposity or fat distribution measures, which are recognised to causally affect blood lipid traits.^45^ Our findings suggest that therapies that do not increase pancreatic uptake of LDL particles while increasing hepatic uptake of LDL particles or precursor VLDL particles might lead to a reduction in risk of both coronary heart disease and type 2 diabetes.

This study has the usual caveats and limitations that apply to mendelian randomisation studies,^46^ and also has additional issues related to the particularities of its design. First, we cannot claim that apoB directly causes outcomes in the first-degree relatives of the UK Biobank participants because the genetically elevated apoB is only an approximation to unconfounded estimates within first-degree relatives. However, the replication of our analyses for lifespan and type 2 diabetes corroborates our findings and therefore strengthens these hypotheses. Second, because first-degree relatives share 50% of their DNA, the effect estimates generated by our genetic instruments have been attenuated through regression dilution. Thus, the magnitudes of effect estimates for coronary heart disease, type 2 diabetes, and lifespan associations using conventional two-sample mendelian randomisation approaches are several times greater than the equivalent estimates obtained using outcomes reported as occurring in first-degree relatives. Third, because outcomes are self-reported, measurement error is likely. For example, an individual who has heart disease might be more likely to report a first-degree relative as also having heart disease. Study participants might also have accidentally answered questions regarding non-blood relatives (eg, adopted individuals, step-parents, or stepsiblings) or half-siblings, which would have weakened effect estimates. Fourth, outcomes related to siblings might represent multiple individuals collapsed into a single trait because individuals were asked about siblings only cumulatively rather than individually. This would dilute effects if more than one sibling has an outcome (eg, if two or more first-degree siblings had coronary heart disease).

The cumulative effect of these limitations probably explains why the effect estimates we report for heart disease (ORs of 1·14 to 1·27) are of considerably smaller magnitude than the approximation to within-individual effect estimates we previously reported using data from UK Biobank and CARDIoGRAMplusC4D (OR 1·7) scaled to the same difference in exposure (1 SD higher apoB in the UK Biobank participants).^12^ A similar difference in the magnitudes of effect is evident between estimates of apoB with risk of type 2 diabetes in first-degree relatives (ORs of 1·17 to 1·33) versus data from DIAMANTE (OR >2). Of note, both estimates for heart disease (using CARDIoGRAMplusC4) and type 2 diabetes (using DIAMANTE) might be inflated by spectrum-bias effects arising from case-control studies that sample cases at the extremes of the phenotypic distribution. Future studies that apply alternative approaches when analysing highly correlated lipoprotein traits might therefore be valuable in corroborating our findings, such as the mendelian randomisation Bayesian model averaging approach.^11^

In conclusion, our evaluation of apoB using outcomes in first-degree relatives identified that higher apoB is detrimental to lifespan and increases the risk of coronary heart disease and type 2 diabetes. Lifestyle and pharmacological approaches to lowering apoB should have widespread beneficial effects, including preventing common diseases and prolonging life.

## Supplementary Material

Supplementary Figures

Supplementary Tables

## Figures and Tables

**Table:** Vital status, age at death, and prevalence of outcomes in first-degree relatives, as reported by UK Biobank participants

	Father (median N=400 687*)	Mother (median N=423 692*)	Siblings (median N=361 816*)
Age at death, years, mean (SD)†	70·9 (13·1)	75·7 (13·3)	NA
Still alive	103 919/450 333 (23·1%)	180 472/454 999 (39·7%)	NA
Alzheimer’s disease or dementia	19 255/399 793 (4·8%)	36 548/423 738 (8·6%)	2094/361 264 (0·6%)
Bowel cancer	23 883/399 920 (6·0%)	22 028/423 135 (5·2%)	8920/361 508 (2·5%)
Breast cancer	NA	35 102/423 458 (8·3%)	16 586/361 809 (4·6%)
Chronic bronchitis or emphysema	46 263/402 389 (11·5%)	25 314/423 692 (6·0%)	10 325/361 823 (2·9%)
Diabetes	38 850/400 687 (9·7%)	40 091/423 892 (9·5%)	31 073/362 826 (8·6%)
Heart disease	133 320/407 557 (32·7%)	85 620/426 240 (20·1%)	37 858/363 542 (10·4%)
High blood pressure	91 242/402 899 (22·6%)	130 948/426 391 (30·7%)	77 059/364 661 (21·1%)
Lung cancer	37 443/401 624 (9·3%)	17 566/423 258 (4·2%)	8199/361 586 (2·3%)
Parkinson’s disease	10 106/399 089 (2·5%)	6998/422 464 (1·7%)	2005/361 199 (0·6%)
Prostate cancer	30 945/399 670 (7·7%)	NA	5952/361 394 (1·6%)
Severe depression	15 430/399 499 (3·9%)	28 351/423 217 (6·7%)	26 368/362 315 (7·3%)
Stroke	62 810/402 616 (15·6%)	60 880/424 977 (14·3%)	12 031/361 925 (3·3%)

Data are % cases unless otherwise stated (numbers of cases and controls for each outcome are presented in appendix 1 table S4). NA=not applicable.

* Value corresponds to the median number of UK Biobank participants reporting any outcome in fathers, mothers, or siblings (details are provided in appendix 1 table S4).

† Age at death for fathers of 341 118 UK Biobank participants and mothers of 273 111 UK Biobank participants.

## Data Availability

Genetic instruments on lipoprotein lipid traits were derived in our previous study (Richardson and colleagues^13^) and are accessible from the online supplementary material of that study. Full GWAS results for these traits are available from the corresponding author. Summary statistics from the GWAS of family outcomes used in this study are publicly accessible on the OpenGWAS platform. Summary statistics for the lifespan GWAS done by Deelen and colleagues^17^ are available online. For GWAS summary statistics on type 2 diabetes excluding UK Biobank, please contact authors AM (anubha@well.ox.ac.uk) or MIM (mark.mccarthy@drl.ox.ac.uk).

## Research in context

### Evidence before this study

Previous observational and mendelian randomisation studies have shown that circulating concentrations of apolipoprotein B (apoB) are of crucial importance to lipid-mediated atherogenesis, manifested as coronary heart disease. We searched PubMed on Feb 19, 2021, with terms including “apolipoprotein B” AND (“mendelian randomization” OR “mendelian randomisation”) AND “longevity”, which yielded no results. A broader search term of “apolipoprotein B” AND “longevity” identified 17 results. Although some studies evaluated associations of plasma concentrations of apoB or genetic variants in *APOB* with lifespan, none explored the causal role of apoB in their analyses or used an approach that quantified its causal role when considering genetically predicted LDL cholesterol or triglyceride levels.

### Added value of this study

In this study, we explored the effects of genetically predicted elevations in apoB on multiple endpoints occurring in first-degree relatives, including lifespan, and sought replication of findings using more conventional methods to exploit the statistical power from data available in large-scale genome-wide association study consortia. We identified that apoB had a deleterious effect on longevity, shortening the lifespan by a period of months to years. Furthermore, apoB caused higher risks of coronary heart disease and stroke in first-degree relatives. Finally, apoB was identified to increase the risk of type 2 diabetes using multivariable mendelian randomisation methods, in contrast to LDL cholesterol, which appeared to the lower risk of type 2 diabetes.

### Implications of all the available evidence

Our findings support apoB as being the major lipoprotein entity implicated in the aetiology of coronary heart disease and stroke and identify that higher apoB decreases lifespan and increases the risk of type 2 diabetes. These findings highlight the crucial role of apoB in causing cardiometabolic disease, which collectively shortens the lifespan.
