# Supplementary material for: Effects of apolipoprotein B on lifespan and risks of major diseases including type 2 diabetes: a mendelian randomisation analysis using outcomes in first-degree relatives
Source: Lancet Healthy Longev. Author manuscript; Available in PMC 2021 Nov 1. (PMC7611924; doi:10.1016/S2666-7568(21)00086-6)
Supplement: Supplementary Figures [file EMS137353-supplement-Supplementary_Figures.pdf]

THE LANCET
Healthy Longevity

Supplementary appendix 2

This appendix formed part of the original submission and has been peer reviewed. We post it as supplied by the authors.

Supplement to: Richardson TG, Wang Q, Sanderson E, et al. Effects of apolipoprotein B on lifespan and risks of major diseases including type 2 diabetes: a mendelian randomisation analysis using outcomes in first-degree relatives. *Lancet Healthy Longev* 2021; published online May 20. https://doi.org/10.1016/S2666-7568(21)00086-6.

Supplementary Materials

Effects of apolipoprotein B on the lifespan and risks of major disease including type 2 diabetes: a Mendelian randomization analysis using outcomes in first-degree relatives

Tom G Richardson, Qin Wang, Eleanor Sanderson et al

(see manuscript for full author list and affiliations)

Table of Contents

Supplementary Figure 1. Schema of how data were used in this study to instrument lipid levels in first-degree relatives. 2

Supplementary Figure 2. Univariable and multivariable Mendelian randomization estimates of apolipoprotein B and risk of endpoints in first-degree relatives........ 3

Supplementary Figure 3. Univariable and multivariable Mendelian randomization estimates of LDL cholesterol and risk of endpoints in first-degree relatives........ 4

Supplementary Figure 4. Univariable and multivariable Mendelian randomization estimates of triglycerides and risk of endpoints in first-degree relatives........ 5

Supplementary Figure 5. Comparison of multivariable Mendelian randomization approaches........ 6

Supplementary Figure 6. Univariable and multivariable Mendelian randomization estimates of apolipoprotein B, LDL cholesterol and triglycerides and risk of type 2 diabetes in DIAMANTE. 7

Supplementary Figure 7. Univariable and multivariable Mendelian randomization estimates of apolipoprotein B, LDL cholesterol and triglycerides and risk of type 2 diabetes in DIAMANTE including adjustment for (B) total and (C) central adiposity........ 8

Supplementary Figure 8. Metabolism of low density lipoprotein under (A) normal physiological conditions and abnormal physiological conditions of: (B) impaired LDL particle uptake by LDL receptor pathway, and; (C) over-production of VLDL particles........ 9

Supplementary Tables 1. – 23. ....please see Excel sheet

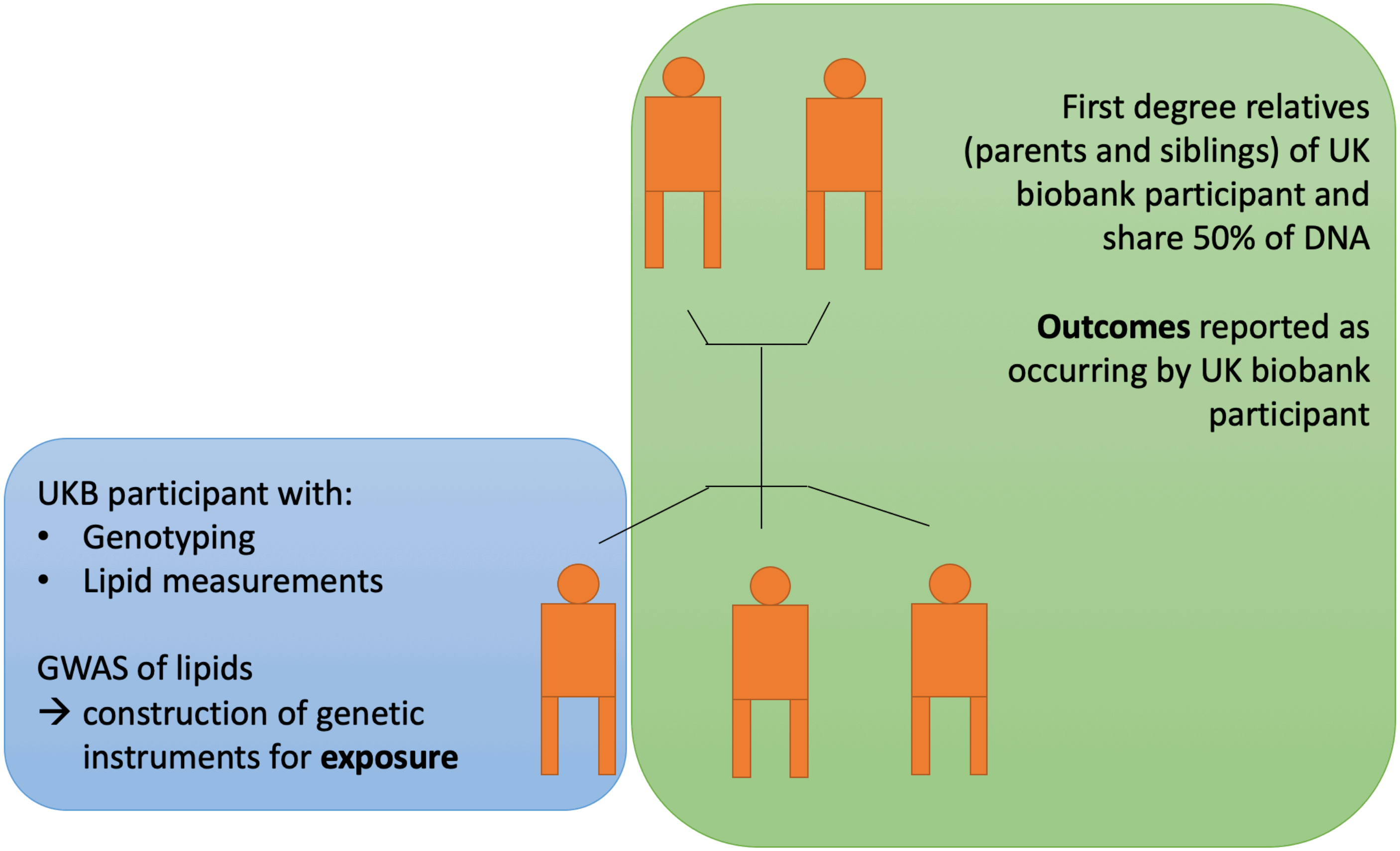

Supplementary Figure 1. Schema of how data were used in this study to instrument lipid levels in first-degree relatives. We constructed genetic instruments in study participants of UKB (blue box). We used these genetic instruments as proxies for levels of lipid in first-degree relatives (green box) and outcomes as reported in these first-degree relatives.

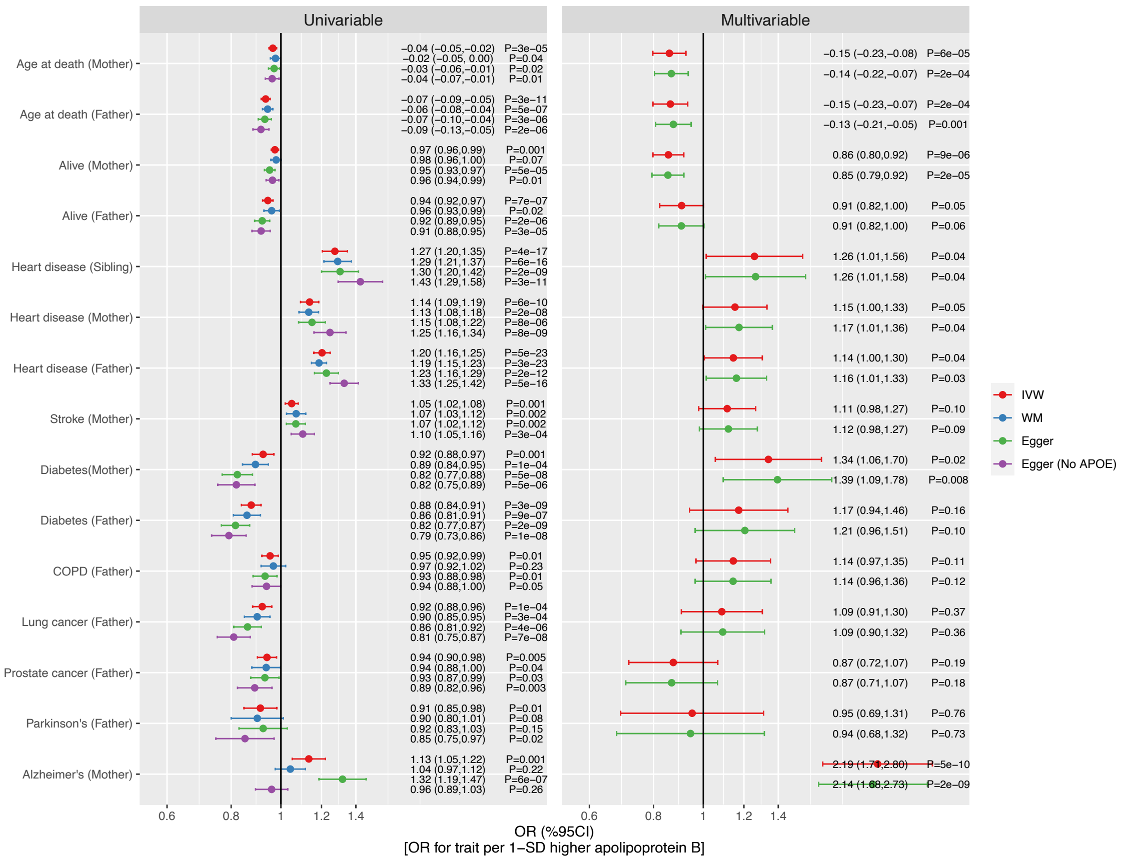

Supplementary Figure 2. Univariable and multivariable Mendelian randomization estimates of apolipoprotein B and risk of endpoints in first-degree relatives. Egger: MR-Egger; Egger (NO APOE): MR-Egger excluding SNPs in/around *APOE;* IVW: inverse variance weighted; MVMR: multivariable Mendelian randomization; UVMR: univariable Mendelian randomization; WM: weighted median. Numerical values for age at death reports beta coefficients (and not odds ratios).

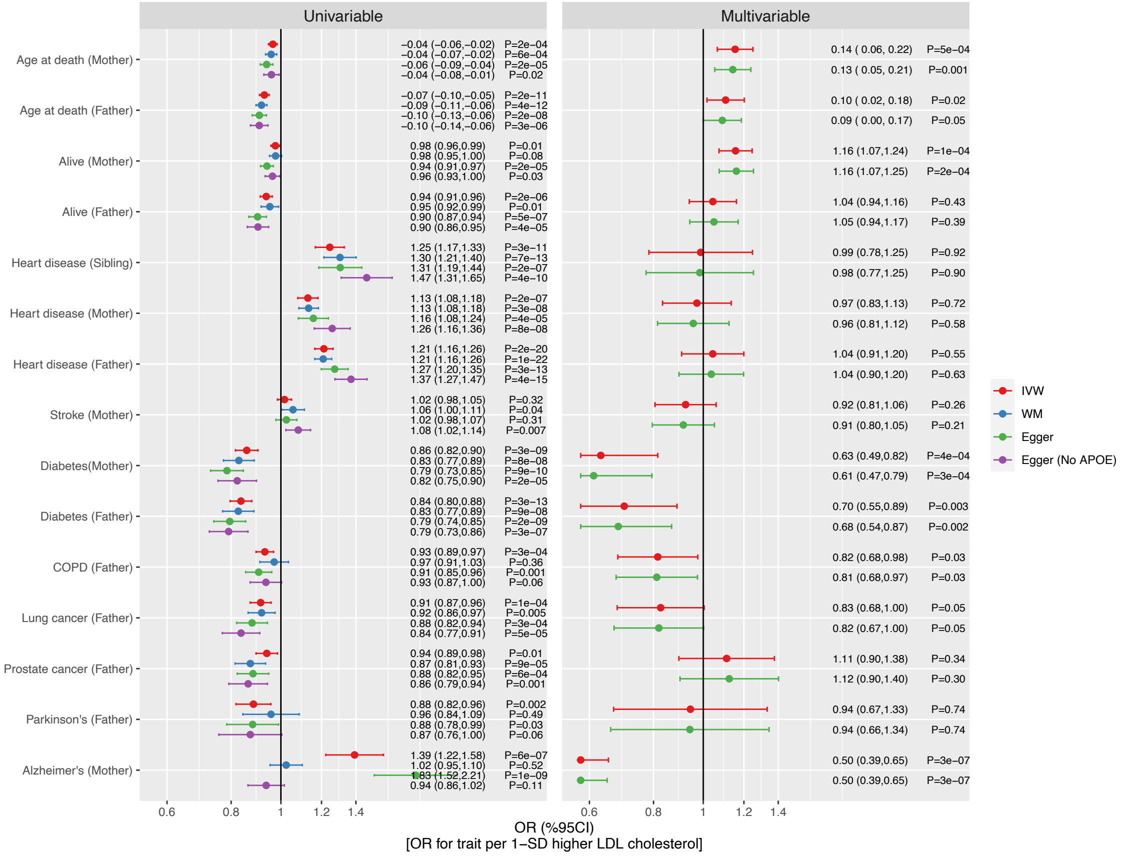

Supplementary Figure 3. Univariable and multivariable Mendelian randomization estimates of LDL cholesterol and risk of endpoints in first-degree relatives.
Egger: MR-Egger; Egger (NO APOE): MR-Egger excluding SNPs in/around APOE; IVW: inverse variance weighted; MVMR: multivariable Mendelian randomization; UVMR: univariable Mendelian randomization; WM: weighted median. Numerical values for age at death reports beta coefficients (and not odds ratios).

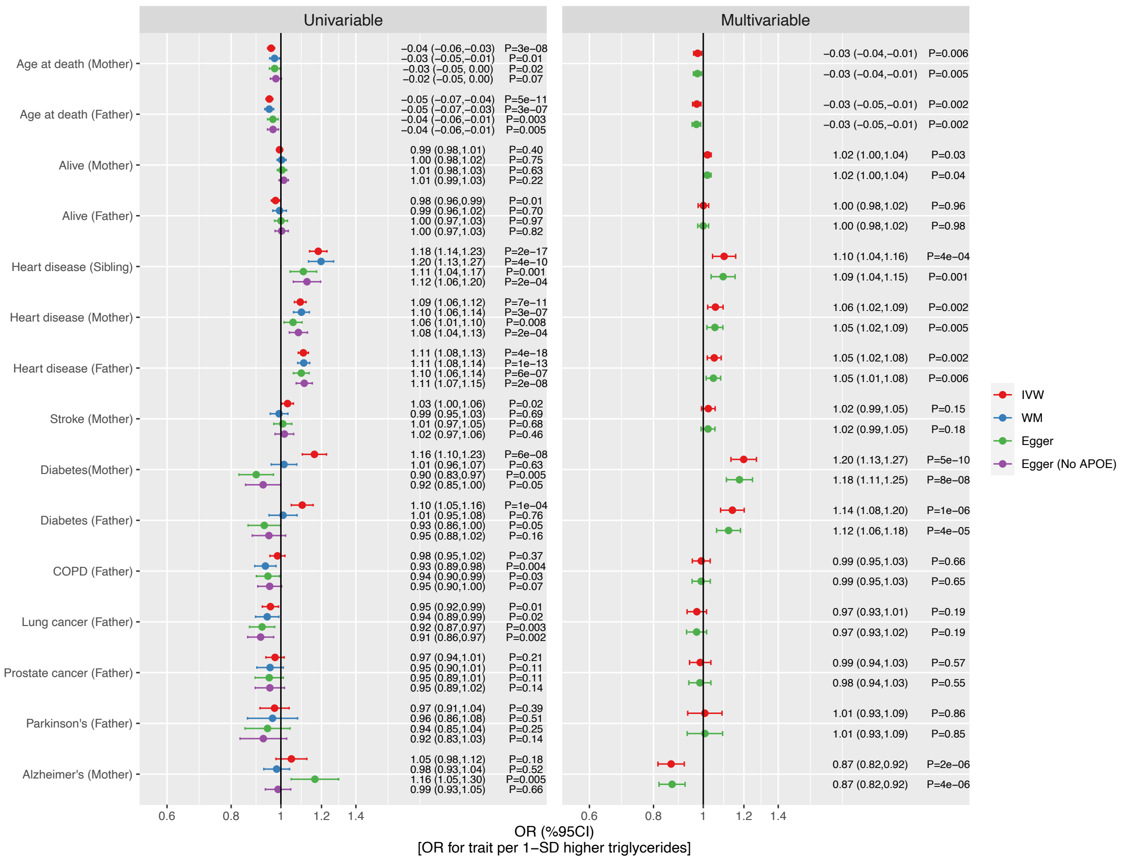

Supplementary Figure 4. Univariable and multivariable Mendelian randomization estimates of triglycerides and risk of endpoints in first-degree relatives.

Egger: MR-Egger; Egger (NO APOE): MR-Egger excluding SNPs in/around *APOE;* IVW: inverse variance weighted; MVMR: multivariable Mendelian randomization; UVMR: univariable Mendelian randomization; WM: weighted median. Numerical values for age at death reports beta coefficients (and not odds ratios).

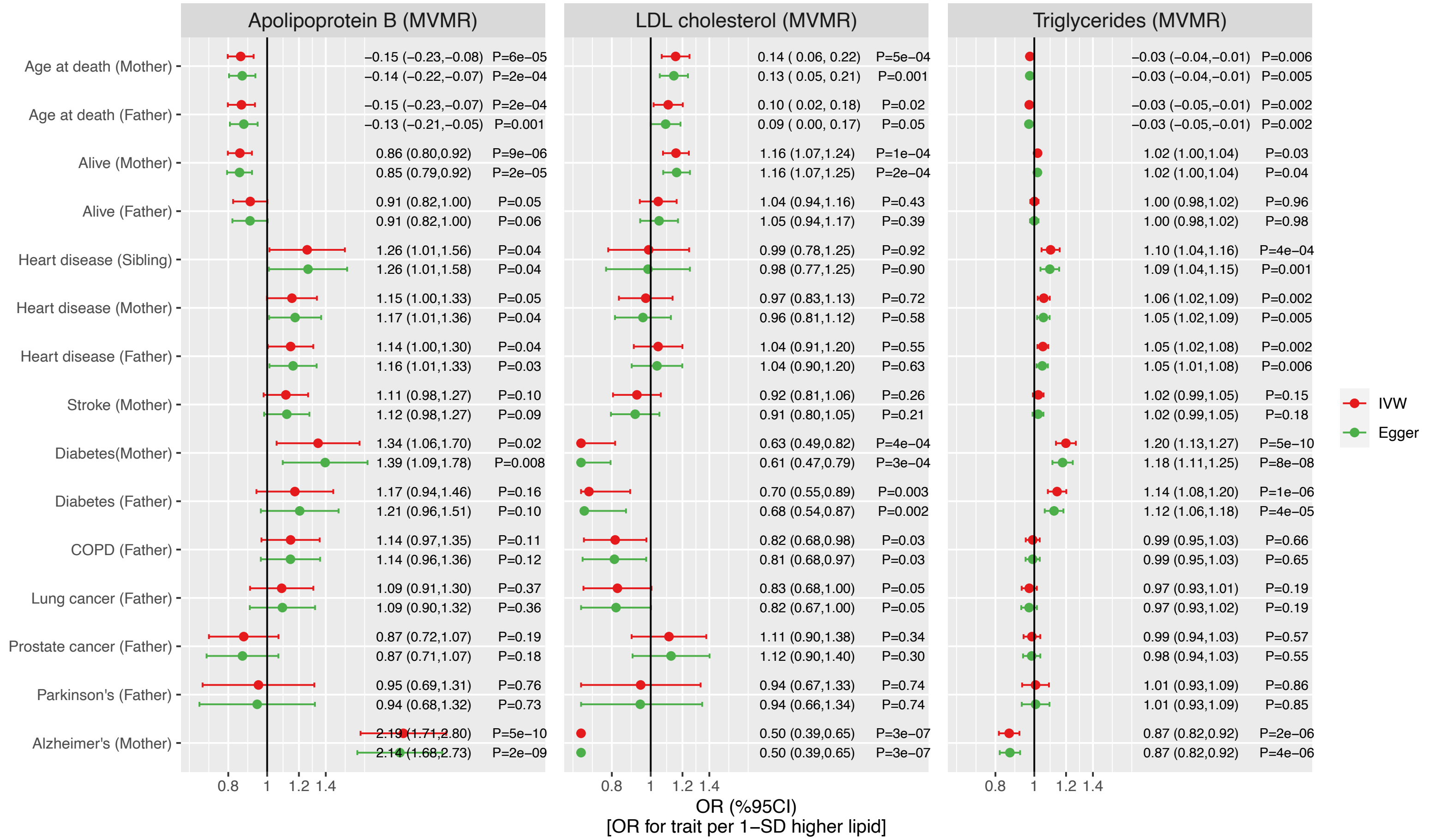

Supplementary Figure 5. Comparison of multivariable Mendelian randomization approaches.

The plot displays estimates from conventional (inverse variance-weighted; IVW) multivariable Mendelian randomization to that of multivariable Mendelian randomization using MR-Egger. Numerical values for age at death reports beta coefficients (and not odds ratios).

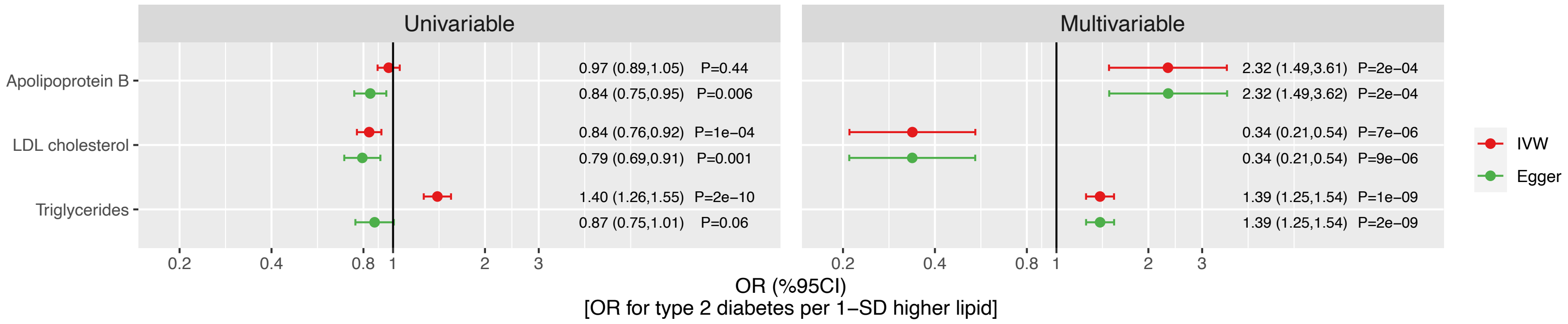

Supplementary Figure 6. Univariable and multivariable Mendelian randomization estimates of apolipoprotein B, LDL cholesterol and triglycerides and risk of type 2 diabetes in DIAMANTE.

Egger: MR-Egger; IVW: inverse variance weighted; MVMR: multivariable Mendelian randomization; UVMR: univariable Mendelian randomization

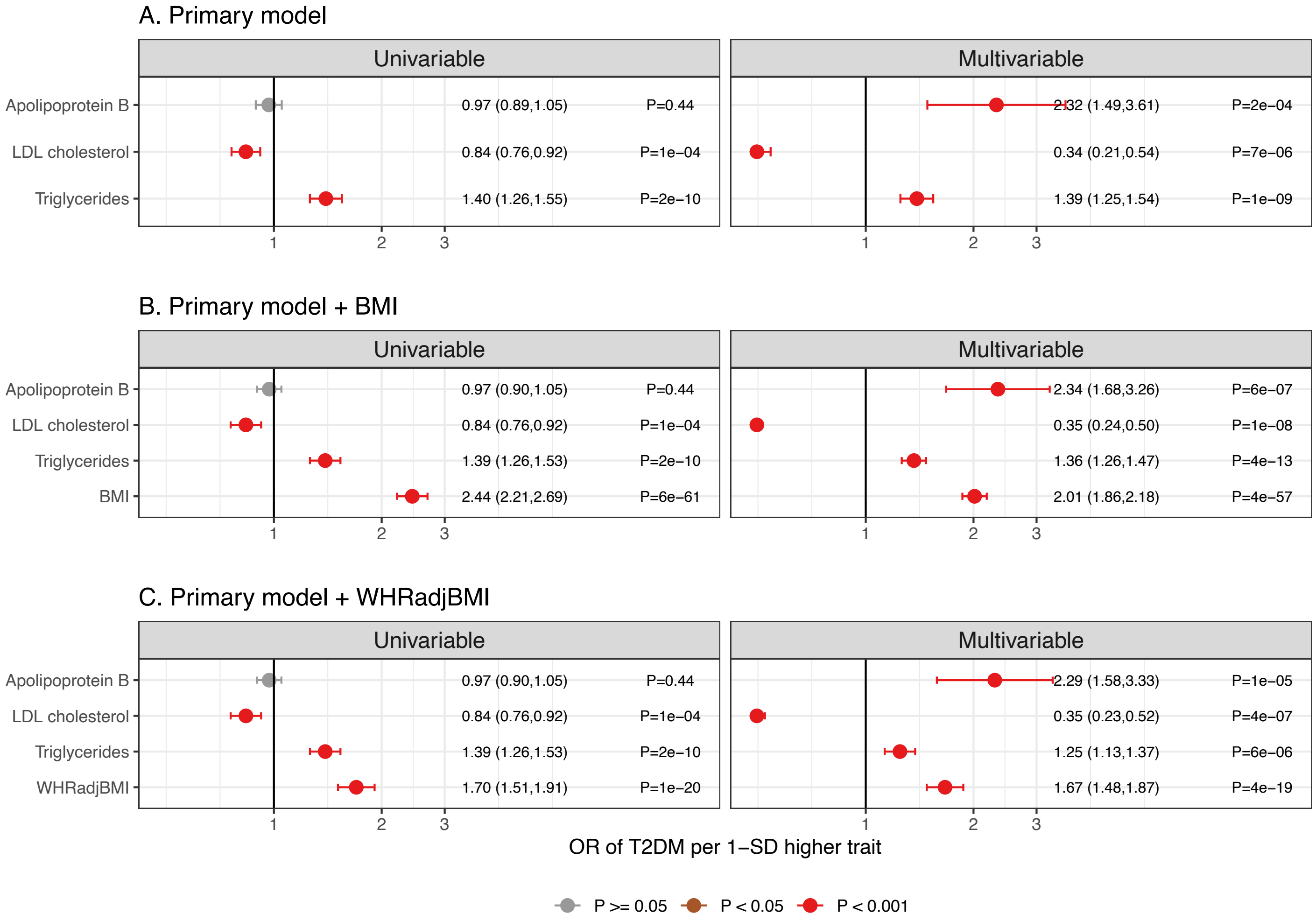

Supplementary Figure 7. Univariable and multivariable Mendelian randomization estimates of apolipoprotein B, LDL cholesterol and triglycerides and risk of type 2 diabetes in DIAMANTE including adjustment for (B) total and (C) central adiposity.
BMI: body mass index, indexing total adiposity. WHRadjBMI: waist-hip ratio adjusted for BMI, indexing central adiposity

Supplementary Figure 8. Metabolism of low density lipoprotein under (A) normal physiological conditions and abnormal physiological conditions of: (B) impaired LDL particle uptake by LDL receptor pathway, and; (C) over-production of VLDL particles.

(A) Normal LDL clearance

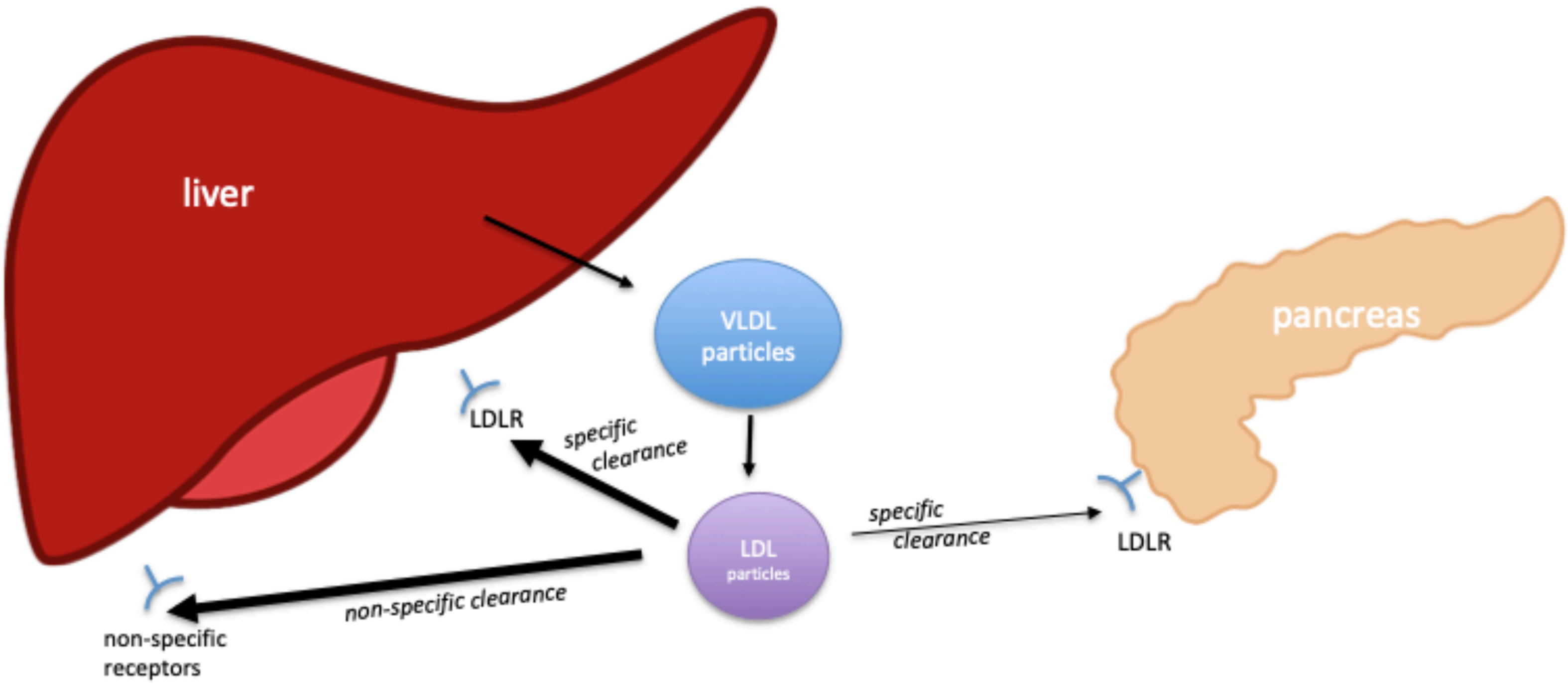

Panel A: metabolism of low density lipoprotein (LDL) under normal physiological processes. The liver synthesizes and secretes VLDL particles which, with removal of triglyceride within them, are transformed into LDL particles. LDL particles are cleared principally by the liver via specific LDL receptors (LDLR), which are concentration-dependent and saturable, and non-specific receptors, which are concentration-independent but non-saturable. As LDLR number decreases, a greater proportion of LDL clearance occurs via non-specific clearance. However, a small portion of LDL particles are cleared by LDLR in peripheral cells, such as the pancreas. Even though the uptake of LDL particles by the pancreas represents a trivial portion of total apoB clearance, we posit that variations in activity of this pathway may lead to altered risk of T2D.

(B) Decreased LDL clearance (e.g. dysfunctional LDL receptor): ↓apoB and ↑LDL-C

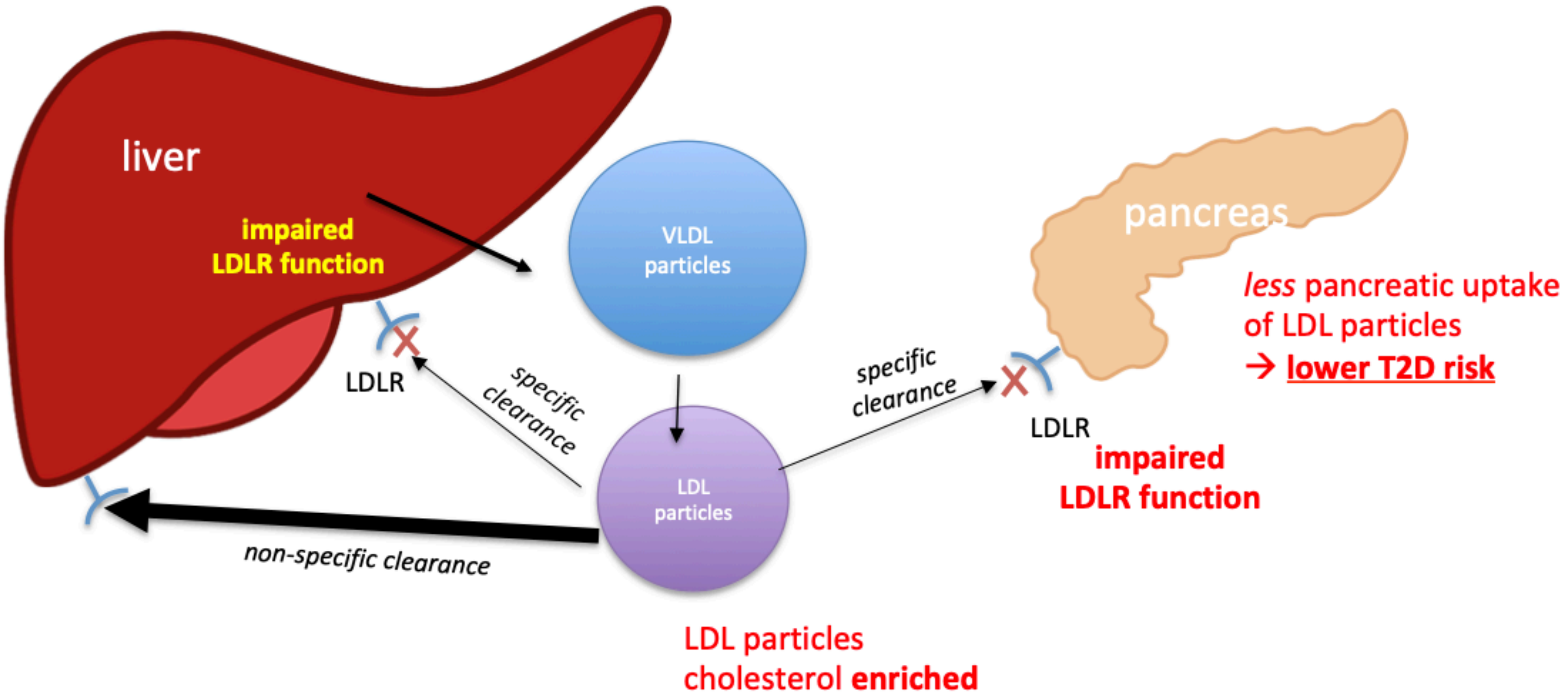

Panel B: impaired LDL receptor (LDLR) function leading to excess circulating LDL particles that are cholesterol enriched. Thus, apoB concentrations in plasma are comparatively lower than LDL-C – this scenario is approximated by our multivariable MR analysis when LDL-C is increased and apoB kept constant. In this circumstance, clearance of LDL from the circulation occurs principally through non-specific pathways. The reduction in LDL clearance through specific pathways (via LDLR) leads to a reduction in uptake of LDL particles by the pancreas. Because LDL uptake in excess can be injurious to beta cell function in the islets of Langerhans within the pancreas, reduced clearance leads to a lower risk of T2D.

(C) VLDL particles over-produced by the liver: ↑apoB and ↓LDL-C

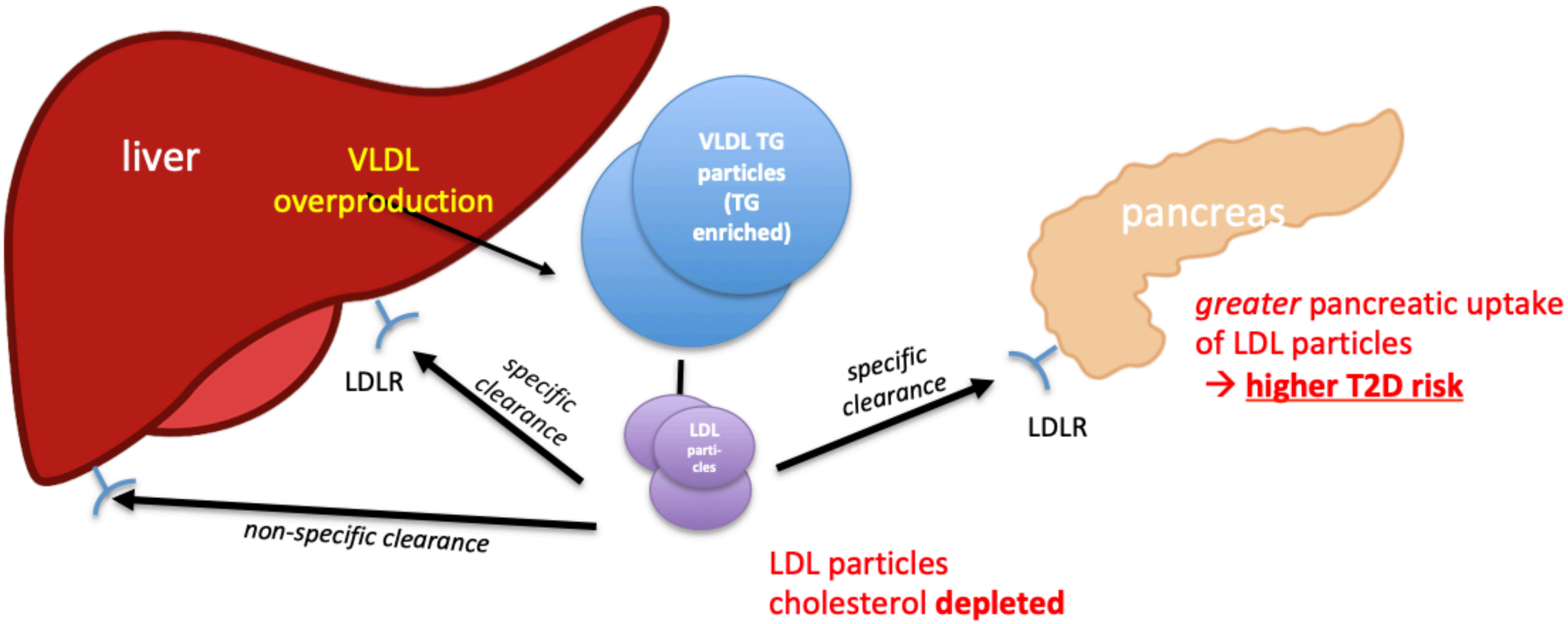

Panel C: VLDL over-production by the liver. This leads to higher circulating LDL particles that are comparatively cholesterol depleted. In other words, circulating apoB concentrations are comparatively higher than LDL-C – this scenario is approximated by our multivariable MR analysis when apoB is increased and LDL-C kept constant. In this scenario, overall LDL clearance is increased and occurs through both LDLR and non-specific pathways. Increased uptake of apoB particles by the pancreas leads to injury with commensurate reduction in function of beta cells and thus a higher risk of T2D.
